# Supplementary material for: Lateralization of CA1 assemblies in the absence of CA3 input
Source: Nat Commun. 2021 Oct 20;12:6114. doi: 10.1038/s41467-021-26389-3 (PMC8528853; doi:10.1038/s41467-021-26389-3)
Supplement: Supplementary file 1 — Supplementary Information [file 41467_2021_26389_MOESM1_ESM.pdf]

# Supplementary Materials for

## Lateralization of CA1 assemblies in the absence of CA3 input

Hefei Guan, Steven J. Middleton, Takafumi Inoue, Thomas J. McHugh*

* Correspondence: thomas.mchugh@riken.jp

This PDF file includes:

Supplementary Fig. 1 to Supplementary Fig. 4

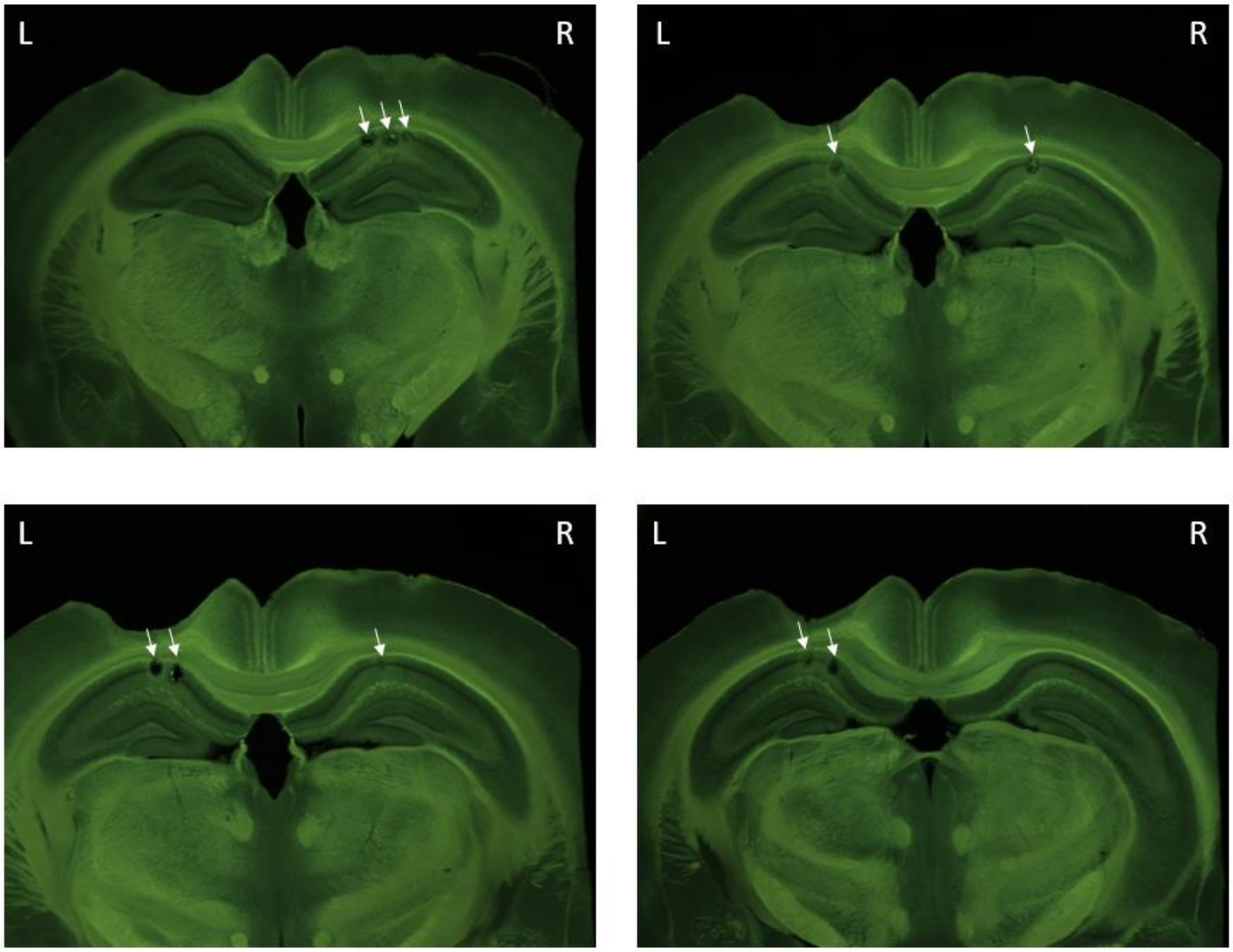


**Supplementary Fig. 1: Histology shows tetrode locations in left and right hippocampal CA1.** The position of individual tetrodes was confirmed via electrolytic lesion. The lesions indicated with arrows show the location of recording tetrodes from equivalent positions in dorsal CA1 across both hemispheres.

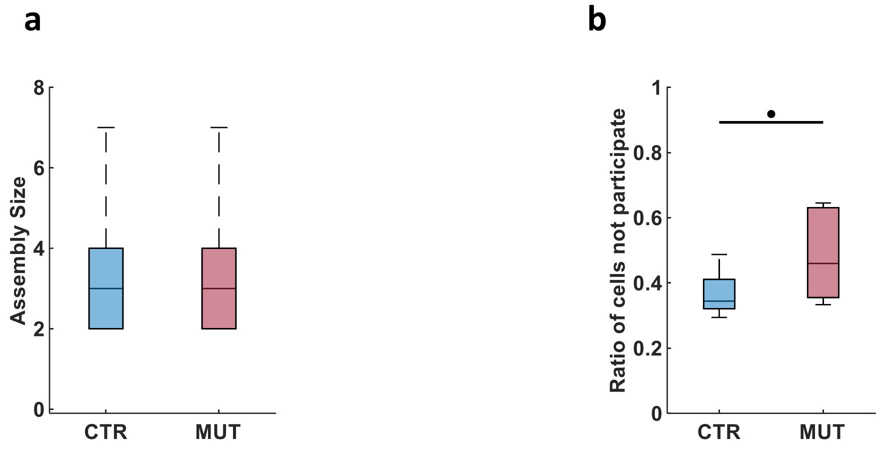


**Supplementary Fig. 2: Assembly size and participation. a** Assembly size was similar in control and mutant mice (CTR: N=4 animals, n=123 assemblies; MUT: N=5 animals, n=121 assemblies; P=0.4811). **b** The fraction of neurons not participating in any assembly was significantly higher in mutant mice (CTR: N=4 animals, n=8 (two directions for each mouse); MUT: N=5 animals, n=10 (two directions for each mouse); P=0.0434). All significant difference in Supplementary Fig.2 were tested with two-sided Wilcoxon rank sum test.

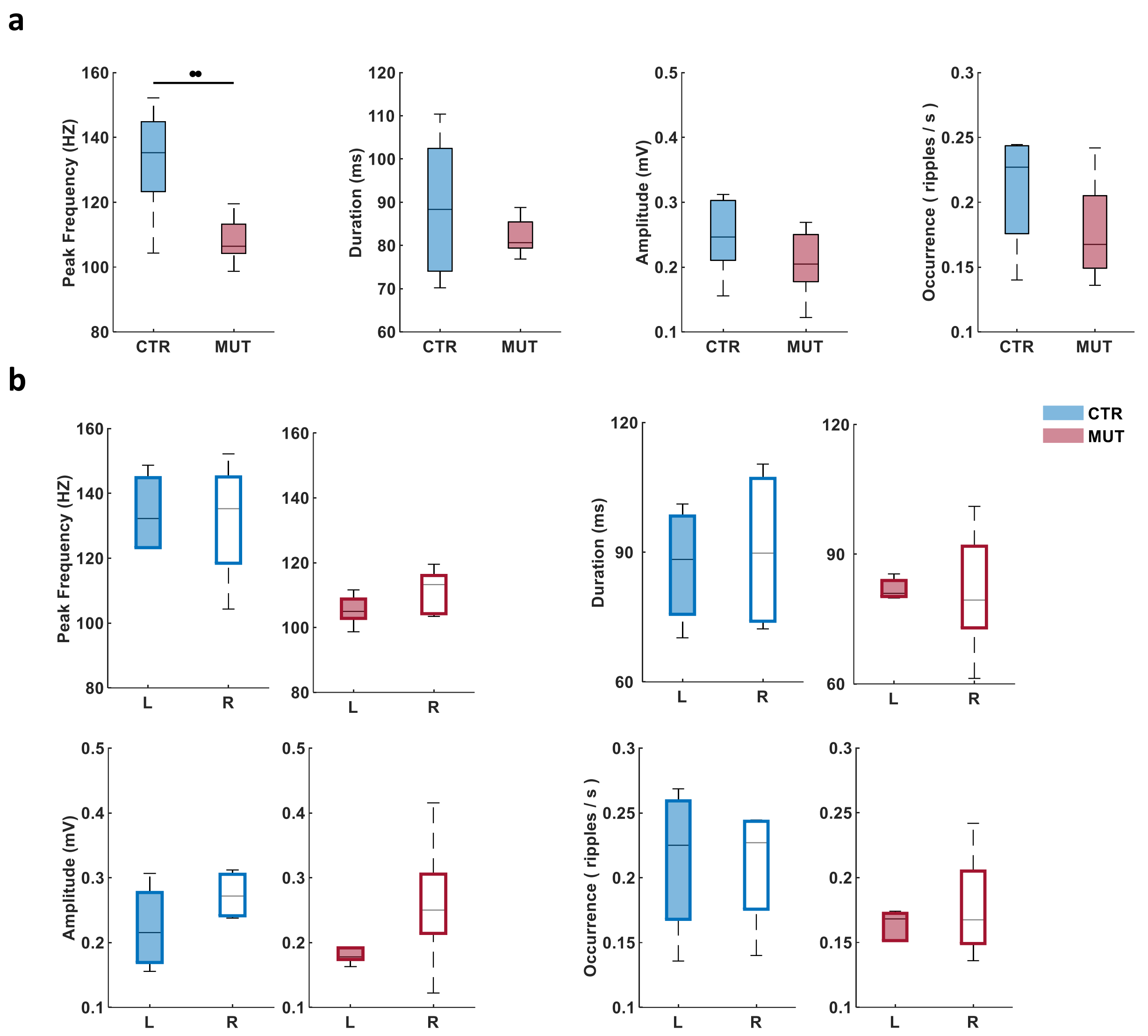


**Supplementary Fig. 3: SWRs properties across genotypes and hemispheres**. **a** Intrinsic SWRs frequency was significantly slower in mutant mice compared to controls, but amplitude, occurrence and duration were similar between genotypes (CTR, N=4 mice, n=8 tetrodes, one from each hemisphere; MUT, N=5 mice, n=10 tetrodes, one from each hemisphere; P (frequency) = 0.0021, P (amplitude) =0.2743, P (duration) = 0.5148, P (occurrence) = 0.0676, two-sided Wilcoxon rank sum test). **b** SWRs properties were similar between left and right CA1 in both genotypes (Filled bars: left hemisphere; open bars: right hemisphere; CTR, N=4 mice, n=8 tetrodes, one from each hemisphere; MUT, N=5 mice, n=10 tetrodes, one from each hemisphere; frequency: P (CTR) = 1, P (MUT)= 0.3095; amplitude: P (CTR) = 0.4857, P (MUT) = 0.1508; duration: P (CTR) = 0.6857, P (MUT)= 0.6905; occurrence: P (CTR) = 0.8857, P (MUT) = 0.8413); significant difference in Supplementary Fig. 3b were tested with LMMs).

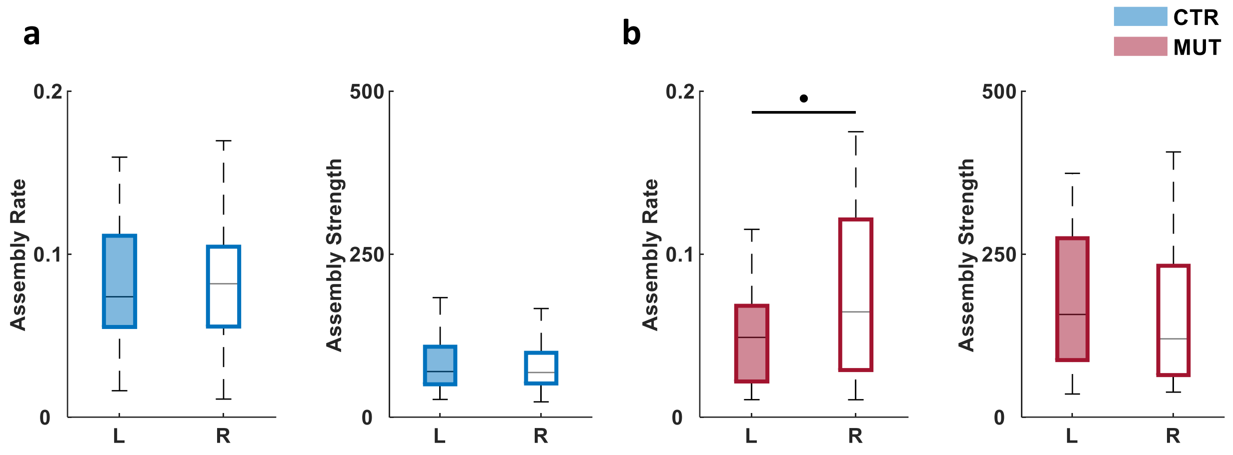


**Supplementary Fig. 4: a** The reactivation of strength and rate of right assemblies and left assemblies were similar in control (CTR, N=4 mice, n (right assemblies with right ripple) = 35, n (left assemblies with right ripple) = 61, P (rate) = 0.7678, P (strength) = 0.0836). **b** Right assembly reactivate faster than left assembly during right ripple in MUT, but strength remained similar (MUT, N=5 mice, n (right assemblies with right ripple) = 46, n (left assemblies with right ripple) = 53, P (rate) = 0.0388, P (strength) = 0.1707). All significant difference in Supplementary Fig.4 were tested with LMMs.
