# Supplementary material for: Lateralization of CA1 assemblies in the absence of CA3 input
Source: Nat Commun. 2021 Oct 20;12:6114. doi: 10.1038/s41467-021-26389-3 (PMC8528853; doi:10.1038/s41467-021-26389-3)
Supplement: Supplementary file 3 — Reporting Summary [file 41467_2021_26389_MOESM3_ESM.pdf]

nature research

Corresponding author(s): Thomas J. McHugh

Last updated by author(s): Sep 27, 2021

Reporting Summary

Nature Research wishes to improve the reproducibility of the work that we publish. This form provides structure for consistency and transparency in reporting. For further information on Nature Research policies, see our Editorial Policies and the Editorial Policy Checklist.

Statistics

For all statistical analyses, confirm that the following items are present in the figure legend, table legend, main text, or Methods section.

n/a	Confirmed	
☐	☒	The exact sample size (*n*) for each experimental group/condition, given as a discrete number and unit of measurement
☐	☒	A statement on whether measurements were taken from distinct samples or whether the same sample was measured repeatedly
☐	☒	The statistical test(s) used AND whether they are one- or two-sided<br>*Only common tests should be described solely by name; describe more complex techniques in the Methods section.*
☐	☒	A description of all covariates tested
☐	☒	A description of any assumptions or corrections, such as tests of normality and adjustment for multiple comparisons
☐	☒	A full description of the statistical parameters including central tendency (e.g. means) or other basic estimates (e.g. regression coefficient) AND variation (e.g. standard deviation) or associated estimates of uncertainty (e.g. confidence intervals)
☐	☒	For null hypothesis testing, the test statistic (e.g. *F*, *t*, *r*) with confidence intervals, effect sizes, degrees of freedom and *P* value noted<br>*Give P values as exact values whenever suitable.*
☐	☒	For Bayesian analysis, information on the choice of priors and Markov chain Monte Carlo settings
☒	☐	For hierarchical and complex designs, identification of the appropriate level for tests and full reporting of outcomes
☒	☐	Estimates of effect sizes (e.g. Cohen's *d*, Pearson's *r*), indicating how they were calculated

Our web collection on statistics for biologists contains articles on many of the points above.

Software and code

Policy information about availability of computer code

Data collection	Cheetah v 5.7.4 (Neuralynx) data acquistion software
Data analysis	SpikeSort 3D v 2.5.0.0 (Neuralynx), Matlab R2019a (MathWorks)

For manuscripts utilizing custom algorithms or software that are central to the research but not yet described in published literature, software must be made available to editors and reviewers. We strongly encourage code deposition in a community repository (e.g. GitHub). See the Nature Research guidelines for submitting code & software for further information.

Data

Policy information about availability of data

All manuscripts must include a data availability statement. This statement should provide the following information, where applicable:

- Accession codes, unique identifiers, or web links for publicly available datasets
- A list of figures that have associated raw data
- A description of any restrictions on data availability

Source data are provided as a Source Data file. Processed data are available at: https://doi.org/10.5281/zenodo.5529405
The custom MATLAB scripts used in this study are available at: https://github.com/HefeiGuan/Guan-et-al-Nat-Comm-2021

Field-specific reporting

Please select the one below that is the best fit for your research. If you are not sure, read the appropriate sections before making your selection.

☒ Life sciences ☐ Behavioural & social sciences ☐ Ecological, evolutionary & environmental sciences

For a reference copy of the document with all sections, see nature.com/documents/nr-reporting-summary-flat.pdf

Life sciences study design

All studies must disclose on these points even when the disclosure is negative.

Sample size	No statistical method was used to pre-determine sample sizes, but we chose sample sizes that exceeded or matched those reported in previous studies with similar methodologies; see manuscript references 2,4-9,11,14,16-18,20,22-30,34,37,38.
Data exclusions	No data was systematically excluded. A limited number of tetrodes were not included for data analysis as they either had no extracellular spike waveforms, or were poorly targeted and not situated in CA1.
Replication	The experimental findings were reliably reproduced across animals in cases where experiments were repeated. Individual animals within genotype showed the same trends as the population. Separate experiments were also internally consistent across figures. The total number of animals and neurons is reported in the figure legends for all experiments.
Randomization	Animals were assigned to groups in a randomized fashion. All other covariates were controlled for including age, sex, experimental diet (doxycycline regime) and timing of experimental procedures, as described in the methods section.
Blinding	Experiments were not performed blind with respect to genotype, the reason for this is that CA3 silencing leads to a very characteristic slowing of the intrinsic frequency of sharp-wave ripples (as previously reported in Nakashiba et al., 2009 and Middleton & McHcrugh, 2016) which is clearly evident to the experimenter during data collection. However, to maximize the objectivity of the study a subset of the data were analyzed blindly (without knowledge of genotype allocation), these results did not differ from the remainder of the data. Further, all data were treated identically across genotypes, with the same criteria applied for the exclusion of putative neurons (as detailed in the methods section), irrespective of genotype.

Reporting for specific materials, systems and methods

We require information from authors about some types of materials, experimental systems and methods used in many studies. Here, indicate whether each material, system or method listed is relevant to your study. If you are not sure if a list item applies to your research, read the appropriate section before selecting a response.

Materials & experimental systems

n/a	Involved in the study
☒	☐ Antibodies
☒	☐ Eukaryotic cell lines
☒	☐ Palaeontology and archaeology
☐	☒ Animals and other organisms
☒	☐ Human research participants
☒	☐ Clinical data
☒	☐ Dual use research of concern

Methods

n/a	Involved in the study
☒	☐ ChIP-seq
☒	☐ Flow cytometry
☒	☐ MRI-based neuroimaging

Animals and other organisms

Policy information about studies involving animals; ARRIVE guidelines recommended for reporting animal research

Laboratory animals	Five male CA3-TeTX transgenic mice and four male control littermates, all aged between 16 weeks and 24 weeks were used in this study. Mice were maintained in a room with a temperature of 22 degree and humidity of 48% on a 12-h light-dark cycle, with lights on from 8:00 to 20:00.
Wild animals	Not used
Field-collected samples	Not used
Ethics oversight	All procedures were approved by the RIKEN Institutional Animal Care and Use Committee.

Note that full information on the approval of the study protocol must also be provided in the manuscript.
